# Organ-On-Chip Technology: The Future of Feto-Maternal Interface Research?

**DOI:** 10.3389/fphys.2020.00715

**Published:** 2020-06-30

**Authors:** Lauren Richardson, Sungjin Kim, Ramkumar Menon, Arum Han

**Affiliations:** ^1^Division of Maternal-Fetal Medicine and Perinatal Research, Department of Obstetrics and Gynecology, The University of Texas Medical Branch at Galveston, Galveston, TX, United States; ^2^Department of Electrical and Computer Engineering, College of Engineering, Texas A&M University, College Station, TX, United States; ^3^Department of Biomedical Engineering, College of Engineering, Texas A&M University, College Station, TX, United States

**Keywords:** organ-on-a-chip, microfluidic lab-on-a-chip, fetal membrane, amniochorion, extracellular matrix

## Abstract

The placenta and fetal membrane act as a protective barrier throughout pregnancy while maintaining communication and nutrient exchange between the baby and the mother. Disruption of this barrier leads to various pregnancy complications, including preterm birth, which can have lasting negative consequences. Thus, understanding the role of the feto-maternal interface during pregnancy and parturition is vital to advancing basic and clinical research in the field of obstetrics. However, human subject studies are inherently difficult, and appropriate animal models are lacking. Due to these challenges, *in vitro* cell culture-based studies are most commonly utilized. However, the structure and functions of conventionally used *in vitro* 2D and 3D models are vastly different from the *in vivo* environment, making it difficult to fully understand the various factors affecting pregnancy as well as pathways and mechanisms contributing to term and preterm births. This limitation also makes it difficult to develop new therapeutics. The emergence of *in vivo*-like *in vitro* models such as organ-on-chip (OOC) platforms can better recapitulate *in vivo* functions and responses and has the potential to move this field forward significantly. OOC technology brings together two distinct fields, microfluidic engineering and cell/tissue biology, through which diverse human organ structures and functionalities can be built into a laboratory model that better mimics functions and responses of *in vivo* tissues and organs. In this review, we first provide an overview of the OOC technology, highlight two major designs commonly used in achieving multi-layer co-cultivation of cells, and introduce recently developed OOC models of the feto-maternal interface. As a vital component of this review, we aim to outline progress on the practicality and effectiveness of feto-maternal interface OOC (FM-OOC) models currently used and the advances they have fostered in obstetrics research. Lastly, we provide a perspective on the future basic research and clinical applications of FM-OOC models, and even those that integrate multiple organ systems into a single OOC system that may recreate intrauterine architecture in its entirety, which will accelerate our understanding of feto-maternal communication, induction of preterm labor, drug or toxicant permeability at this vital interface, and development of new therapeutic strategies.

## Introduction

The challenges and limitations in studying complex human organ or organ systems have spurred interdisciplinary collaboration to develop advanced human cell culture platforms that better mimic the structure and functions of human organ systems for studying their physiological and pathological processes. The combination of microfabrication, microfluidics, and induced pluripotent stem cell (iPSC) technologies has provided many physiological models that better mimic human anatomy, functions, and responses more accurately as seen *in vivo* than traditional 2D cell culture and some animal models (Liu et al., 2018; Sances et al., 2018; Ramme et al., 2019; Jagadeesan et al., 2020). These platforms, termed organ-on-chips (OOCs) or also called microphysiological systems (MPSs), can provide compartmentalized chambers that enable culturing and organizing cellular, extracellular matrices (ECMs), and other microenvironmental layers within these compartments (Huang et al., 2017; Mondrinos et al., 2017; Pasman et al., 2018), while still providing avenues for cellular signals, and sometimes even cells themselves, to migrate between the compartments through interconnected fluid paths (Ren et al., 2017; Richardson et al., 2019b). These systems allow researchers to test many different biomolecular factors under a more physiologically relevant *in vitro* environment, leading to a better understanding of human physiology through gathering significant amounts of data much faster and potentially much more cost-effectively (Huh, 2015; Maschmeyer et al., 2015; Gori et al., 2016; van der Helm et al., 2016; Bein et al., 2018; Guo et al., 2018; Carvalho et al., 2019). In the United States, significant investments made by the Defense Advanced Research Project Agency (DARPA) and the National Institutes of Health (NIH, especially the National Center for Advancing Translational Sciences) have spurred this area in the past decade. Currently, many pharmaceutical and biotechnology companies, as well as many government entities such as the NIH, the Food and Drug Administration (FDA), and Environmental Protection Agency (EPA) are actively interested in utilizing validated OOC systems to conduct pharmaceutical and chemical toxicity studies as well as collect pre-clinical data due to their ability in better replicating human physiology and responses (Capulli et al., 2014; Esch et al., 2015; Konar et al., 2016; Balijepalli and Sivaramakrishan, 2017).

While the goal of OOC technology is not to build whole living organs, these OOC systems are designed to establish a minimally functional unit of organ systems that can better recapitulate certain aspects of human physiology in *in vitro* model systems. Over the past decade, several studies have ushered in the era of OOC technology by replicating organs such as the heart (Zhang et al., 2015, 2016; Jastrzebska et al., 2016; Wan et al., 2018), lung (Huh, 2015; Konar et al., 2016; Shrestha et al., 2020), intestine (Kim et al., 2012; Bein et al., 2018; Guo et al., 2018), liver (Maschmeyer et al., 2015; Esch et al., 2016; Gori et al., 2016; Ramme et al., 2019), kidney (Maschmeyer et al., 2015; Wilmer et al., 2016; Ashammakhi et al., 2018; Ramme et al., 2019), skin (Maschmeyer et al., 2015; Materne et al., 2015; Mori et al., 2017; van den Broek et al., 2017; Bal-Ozturk et al., 2018), blood–brain barrier (BBB) (van der Helm et al., 2016; Jeong et al., 2018; Jagadeesan et al., 2020), bone (Hao et al., 2018; Truesdell et al., 2020), eye (Dodson et al., 2015; Bennet et al., 2018; Haderspeck et al., 2019), and ovary (Nagashima et al., 2018; Weng et al., 2018), to name a few. For a more thorough review of currently available OOCs, refer to these reviews (An et al., 2015; Esch et al., 2015; Balijepalli and Sivaramakrishan, 2017; Low and Tagle, 2017; Kimura et al., 2018). Although they each started with simplistic models, each of these platforms has now been advanced to adapt novel physiologically relevant functions such as cellular contractions (i.e., heart, lung, and eye) (Huh, 2015; Qian et al., 2017; Seo et al., 2019), drug synthesis and excretion (i.e., liver and kidney) (Paoli and Samitier, 2016; Deng et al., 2019), barrier functions (i.e., skin and brain) (Jeong et al., 2018; Mieremet et al., 2019), dynamic flow of blood, air, or fluid interfaces (i.e., heart, lung) (Ribas et al., 2016; Artzy-Schnirman et al., 2019), and even co-culture with bacterial microbiomes (i.e., intestine) (Jalili-Firoozinezhad et al., 2019) in order to replicate the human organ systems of interest. In addition, multiple organ chips can be integrated, either physically through tubing or microfluidic channels or virtually by sending effluents from one OOC to another OOC, to create *in vitro* models of interconnected organ systems, with the ultimate goal of mimicking the entire human physiology (Maschmeyer et al., 2015; Materne et al., 2015; Kimura et al., 2018; Ramme et al., 2019).

From a basic science perspective, microfabricated microfluidic OOC platforms that replicate the microarchitecture of complex organ systems have opened up new experimental procedures to researchers that can utilize such platforms to study contributions of individual cells, cell–cell and cell–ECM interactions, and various biochemical factors to normal organ functions, and also how such functions are influenced by various factors that can be experimentally applied. Furthermore, these models can be extended to mimic a pathologic state, study disease physiology, and mechanisms of action, highlighting the usefulness of these devices in advancing our understanding of human physiology. The recent investments made by the US NIH (NCATS and many other NIH institutes) focusing on the development and utilization of disease OOCs are expected to advance this field further (Ronaldson-Bouchard and Vunjak-Novakovic, 2018; Ouchi et al., 2019; Park et al., 2019; Taylor et al., 2019; Vatine et al., 2019; Wang et al., 2019; Zhao et al., 2019).

From a clinical perspective, *in vitro* cell culture techniques and *in vivo*, small and large animal models, have been the backbone to collect pre-clinical data (Umscheid et al., 2011). The ever-increasing cost of new drug development, stemming in large part due to the large number of drugs that fail at the clinical trial phases due to toxicity or lack of efficacy, or which show conflicting results in animal models, have led to researchers beginning to look for methods that can better predict the toxicity and efficacy of potential drug compounds. OOCs are poised to fill this gap by providing physiologically relevant platforms for better modeling health and disease states of human organ systems. Currently, a variety of OOC platforms are being used in these settings, to model processes such as: (1) mode and mechanism of action, (2) pharmacokinetics and pharmacodynamics, (3) toxicity, (4) efficacy, and (5) dose–response (Luni et al., 2014; Abaci and Shuler, 2015; Esch et al., 2015; Ribas et al., 2016; Wilmer et al., 2016; Balijepalli and Sivaramakrishan, 2017; Low and Tagle, 2017; Bal-Ozturk et al., 2018; Jodat et al., 2018; Kimura et al., 2018; Artzy-Schnirman et al., 2019; Haderspeck et al., 2019; Mittal et al., 2019; Pemathilaka et al., 2019b; van den Berg et al., 2019).

**FIGURE 1:**
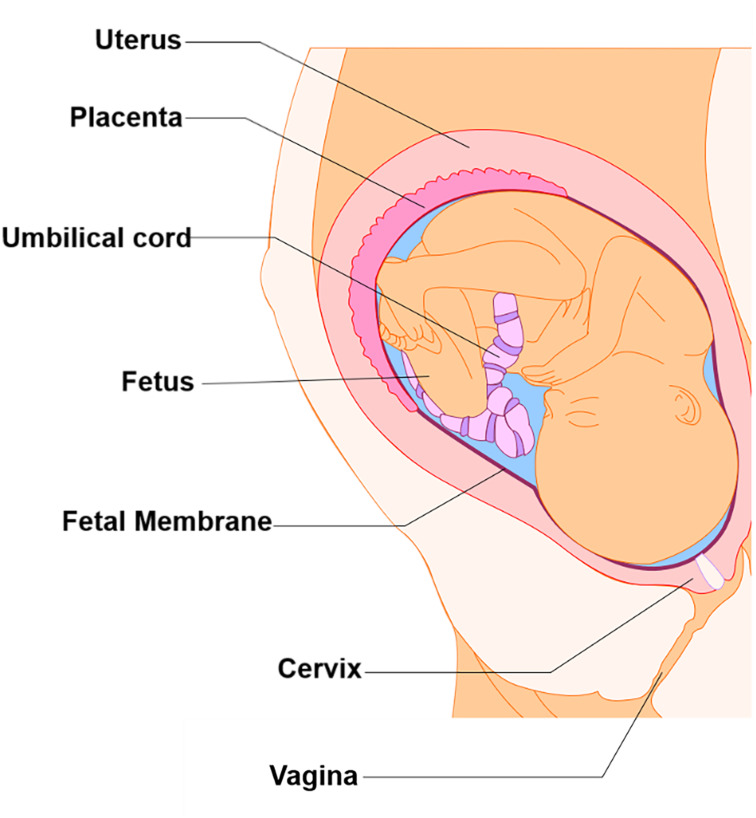
Intra-uterine tissue anatomy. An illustration of the anatomy of the intra-uterine tissue broken down into maternal and fetal components. Maternal tissues comprise of the uterus (i.e., Myometrium), cervix, and vagina, while the fetal tissues include the placenta, umbilical cord, fetus, and fetal membranes.

**FIGURE 2:**
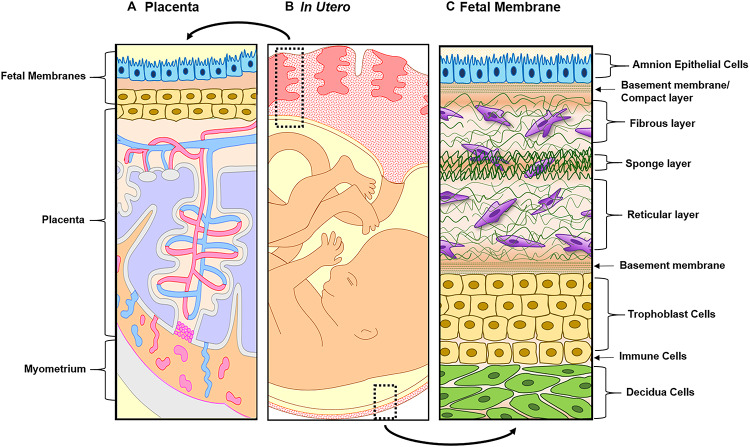
Illustration of both feto-maternal interfaces *in utero*. **(A)** The left side represents the placenta, the site of nutrients, oxygen, and waste exchange for the growing fetus. The placenta is attached to the maternal side by the decidua basalis next to myometrium (gray) and the fetal side through the fetal membranes (amnion in blue and chorion in yellow). This image highlights the tertiary chorionic villi and arteries (red) and veins (blue), respectively. **(B)** Overview of the two feto-maternal interfaces in relation to the fetus. The top box outlines the cross-section of the placenta and the bottom box outlines the cross-section of the fetal membrane. **(C)** The description starts from the innermost layer (amnion) and ends at the maternal decidua. Amnion epithelial cells (blue) are connected to the first layer of the ECM called the basement membrane/compact layer (green strips). The fibroblast (top), spongy (middle), and reticular layers (bottom) follow, containing amnion and chorion mesenchymal cells (purple). The chorion (yellow) is connected to the ECM through a basement membrane (green stripes and is made up of two types of cells: chorion laeve cells and chorion trophoblast cells. The chorion interfaces with the maternal decidua (green), connecting the fetal layers to the maternal compartments of the uterus.

Although many fields have seen the development and advancement of OOC platforms to model physiological and pathological states of their organ systems of interest, the area of obstetrics is only now applying this emerging technique to study pregnancy and preterm birth (Blundell et al., 2016, 2018; Lee et al., 2016; Gnecco et al., 2017; Pemathilaka et al., 2019a, b; Richardson et al., 2019a; Yin et al., 2019). Unlike other single organ model systems, pregnancy introduces new fetal-derived organs within the mother’s uterine cavity (i.e., placenta, umbilical cord, fetus, and fetal membranes) for a period of 9 months (Figure 1) (Menon et al., 2016; Richardson et al., 2018b). These new organs play an essential role in pregnancy maintenance, development, and induction of parturition. Two of these fetal-derived organs, namely, the placenta (Figures 2A,B; top dotted box) and fetal membrane (also known as amniochorion membrane or placenta membrane) (Figures 2B,C; bottom dotted box), create the feto-maternal interface throughout gestation; (1) between placenta and decidua basalis and (2) between fetal membranes and decidua parietalis. The decidua basalis is where implantation takes place and the basal plate is formed. This can be subdivided into a zona compacta and a zona spongiosa, where the detachment of the placenta takes place following birth. The decidua capsularis lies like a capsule around the chorion, while the decidua parietalis remains on the opposite uterus wall. Around the fourth month of gestation, the fetus is so large that the decidua capsularis comes into contact with the decidua parietalis. The merging of these two deciduae causes the uterine cavity to obliterate and forms the two feto-maternal interfaces. The placenta is comprised of the decidua basalis connected to the myometrium (gray), tertiary chorionic villi and intervillous space (light purple), and the reflective amniochorion membrane (blue and yellow cells). The placenta plays a critical role in maintaining pregnancy by regulating maternal metabolism, endocrine and immune functions in addition to providing blood flow [arteries (blue) and veins (red)], nutrients, and oxygen to the fetus, while removing waste products such as carbon dioxide (Jabareen et al., 2009; Mauri et al., 2013; Edey et al., 2018) (Figures 2A,B). The fetal membrane, which surrounds the baby throughout gestation, provides essential immune, endocrine, and mechanical functions (Figures 2B,C) that maintain pregnancy (Jabareen et al., 2009; Boldenow et al., 2013; Mauri et al., 2013, 2015; Perrini et al., 2015; Menon, 2016; Menon et al., 2016, 2017; Sato et al., 2016; Edey et al., 2018; Shah et al., 2019). They are comprised of two epithelial layers, the amnion (blue) and chorion (yellow), separated by an ECM containing mesenchymal cells (purple). The chorion layers are connected to the first layer of the decidua, termed the parietalis (green). As reviewed by Menon and Moore recently, this is one of the least studied intrauterine organs as it is often considered as an extension of the placenta or a dead tissue upon delivery (Menon et al., 2016). At term or preterm, redox imbalances within the intrauterine cavity induce a telomer-dependent, p38MAPK-mediated, cellular senescence in the amnion epithelial cells (AECs) (Figure 2C), which propagate damage-associated molecular patterns (DAMPs) and senescence-associated secretory phenotypes (SASPs) to the maternal decidua (Figure 2C), contributing to the initiation of labor (Menon et al., 2013, 2016; Behnia et al., 2015; Polettini et al., 2015; Sheller et al., 2016; Dixon et al., 2017; Hadley et al., 2018). Fetal membrane-derived signals are one of the essential fetal-derived messages of parturition at term and preterm (Menon et al., 2018; Menon, 2019). In this review, we will focus on the fetal membranes by (1) highlighting the conventionally utilized techniques to study their physiology and contribution to parturition, (2) discuss major design elements of OOCs that enable multi-layer co-cultivation of cells, (3) introduce recently developed feto-maternal interface OOC (FM-OOC) models, and (4) provide a perspective on the future development and impact of pregnancy-related OOCs in the field of obstetrics.

## Current Methods to Study the Feto-Maternal Interfaces and their Limitations

Fetal membranes (structure detailed in Figure 2C) are different from the placenta in terms of their origin, structure, cell types, and functions (Gude et al., 2004; Menon et al., 2018; Richardson et al., 2018b). Not surprisingly, due to these differences, the *in vivo* and *in vitro* models used to study these two distinct feto-maternal interfaces are also unique. Below we will discuss: (1) the similarities and differences between human anatomy and commonly used large and small animal models and (2) the advantages and limitations to current *in vitro* and *ex vivo* techniques.

### Limitations of Animal Models and Current *in vitro* and *ex vivo* Culture Techniques

#### Animal Models

**FIGURE 3:**
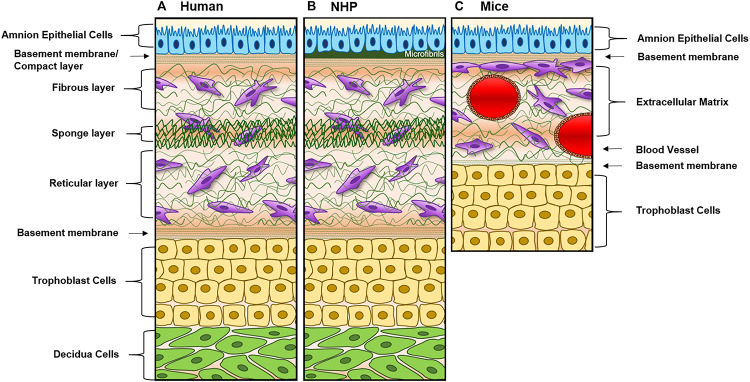
Diagram of fetal membranes anatomical differences between species. **(A)** Illustration representing the human fetal membrane. The human fetal membrane, or feto-maternal interface, starts with the innermost layer (amnion) facing the amniotic cavity and ends with the maternal decidua. Within the intrauterine cavity, the amnion epithelial cells (AECs) connected to the basement membrane (green stripes) are bathed in amniotic fluid (yellow) and comprise the first layer. Below AECs, the ECM is comprised of compact, fibrous, spongy, and reticular layers, all containing mesenchymal cells derived from the amnion and chorion (purple). Chorion trophoblast cells (CT; yellow) are attached to the ECM via another layer of the basement membrane on its apical side and to the maternal decidua (green) on its basal side. **(B)** Schematic of non-human primate (NHP) fetal membranes, currently the best animal model used in the field. It is almost identical to human fetal membranes; however, specific to NHPs, the AEC layer interfaces with a thick, fibrous, collagen layer termed “microfibers” (Owiti et al., 1989) before the basement membrane/compact layer of the ECM. **(C)** The amniotic sac of a mouse is comprised of two epithelial layers; amnion epithelial monolayer (blue) and a multilayer of chorion trophoblast cells (yellow). Between these layers, loose collagen fibers support mesenchymal cells (purple) and maternal blood vessels in the ECM. This tissue does not contain a maternal interface (i.e., decidua) as other mammalian models.

Large and small animal models [i.e., non-human primates (NHP) and mice] are often used for fetal membrane studies. However, differences in pregnancy physiology and structure and uterine environment and cost to conduct studies often hamper the use of these models. NHP models most closely resemble the human fetal–maternal interface (Figure 3A), only deviation being the addition of densely packed fibrous layer covering the basal side of the amnion epithelium termed “microfibrils” (Figure 3B; dark green). These microfibrils could hinder communication between the AEC and ECM layers, which in humans have been shown to be vital for pregnancy maintenance and labor signaling at term. Apart from this, NHPs are anatomically and functionally the most similar to humans, and serve as a reproducible model that enables longitudinal testing of experimental outcomes (Table 1). However, the limitations of this large animal model lie in: (1) the cost of each animal and future housing expenses, (2) handling difficulties, and (3) the need for a proper facility to conduct experiments (Table 1). While NHPs are the gold standard for large animal cytotoxicity studies, murine models such a CD1 and C57BL/6 mice are commonly utilized for small animal pre-clinical experiments. This is primarily due to the fact that murine experiments are easy to conduct, cost-effective, and have a short gestation (Table 1). Although valuable information can be gained from such models, the vast differences in anatomy (i.e., vasculature and maternal layers), and the induction of parturition (i.e., luteolysis), limit their use (Table 1). Regarding fetal–maternal interface anatomy, during murine gestation, the “amniotic sac” develops and surrounds each fetus, mimicking the fetal membrane. The amniotic sac is comprised of two epithelial layers; an amnion epithelial monolayer (blue in Figure 3C) and a multilayer chorion trophoblast cells (yellow in Figure 3C). Between these layers, loose collagen fibers support mesenchymal cells (purple in Figure 3C) and maternal blood vessels in the ECM (Figure 3C). This tissue does not contain a maternal interface (i.e., decidua), other than maternal blood flow, like other mammalian models. These anatomical differences are the biggest hindrance when conducting physiological or pharmaceutical related experiments in murine systems.

**TABLE 1:** Benefits and limitation of *in vivo* and *in vitro* fetal membrane methodology.

	**Model**		**Sub-type**	**Benefits**	**Limitations**
***In vivo***	Murine			• Cost-effective • Easy to handle • Longitudinal sample available	• Anatomical differences • Parturition initiation differences • Small sample size per pup • Large pup number • Endocrine differences
	Non-human primate			• Anatomical similarities • Parturition initiation similarities • Longitudinal sample availability • Reliable large animal model • Small fetal number	• Not cost-effective • Expensive to maintain Hard to handle
	Human			• Preferred primary source • Correct anatomy • Parturition initiation standard	• Clinical trial difficult • Hard to acquire samples • Require infrastructure to store samples properly • Difficult to collect longitudinal samples
	2D cell culture	Sing cell type culture	Primary cells	• Human cells • Physiologically relevant • Maintain *in vivo* characteristics	• Patient-to-patient variability • Difficult to culture • Only studying a part of the whole tissue
***In vitro***	2D cell culture Transwell co-culture	Sing cell type culture Multi-cell type mixed culture	Immortalized human cells	• Derived from human cells • Sustainable in culture	• Have to prove physiological relevance • *n vivo* characteristics lost • Only studying a part of the whole tissue
			iPSC	• Sustainable in culture • Universal lab standard	• Many cell types do not yet exist • Have not been optimized for the feto-maternal interface • Only studying a part of the whole tissue
				• Ability to study cell–cell interactions	• All cell types are mixed together • Hard to determine signal initiation
		Co-culture		• Ability to study cell–cell or cell–collagen interactions • Ability to study signaling propagation and barrier function	• Difficult to culture cells on both sides the membrane • Low throughput • High signal-to-noise ratio • Cell type limitations • Difficult to expand beyond two cell type co-culture
	3D cell culture	Spheroids		• 3D growths of cells • Better maintain *in vivo* characteristics • High throughput • Mixed co-culture possible	• Small cell number leads to limited phenotypic assays • Time-consuming to form • Not uniform • May not organize properly into proper organ structure
	3D cell culture Whole tissue	Cell sheets		• Full amnion layer • Can be cultured longer than amnion explants Easy to image	• Very fragile • Only mimic the amnion layer • Lacks uniformity •
		3D cell printing		• Can recreate multiple feto-maternal interface layers • Proper tissue organization • Uniform production	• Can apply unwanted shear stress to cells during printing • Time-consuming to characterize for each cell type and ECMs to be printed Have not been demonstrated for feto-maternal interface yet
		Explant culture		• Correct tissue organization • Mimics *in vivo* signaling	• Hard to acquire samples • Require infrastructure to store samples properly • Only culturable for up to 72 h or less
					

A full list of the advantages and limitations of each model described in this section can be found in Table 1, and the anatomical differences can be seen in Figure 3.

#### Cell Sources for *in vitro* Models

The standard in fetal membrane research is maintaining membranes as explants *in vitro* (Fortunato et al., 1994) or culturing primary cells (Menon et al., 2013; Sheller et al., 2016; Hadley et al., 2018; Jin et al., 2018; Richardson et al., 2020b), both obtained from discarded human fetal membranes dissected after placental delivery. Though this brings in patient-to-patient variability (Table 1), these approaches maintain some of the *in vivo* characteristics such as cytoskeletal organization (Menon et al., 2018; Richardson et al., 2018b, 2020b), endocrine and paracrine signaling (Myatt and Sun, 2010; Behnia et al., 2015), inflammatory responses (Menon et al., 2009; Noda-Nicolau et al., 2016), as well as immune regulatory factors (Fortunato et al., 1998, 2001). Protocols documenting amnion (i.e., AEC and AMC) cell isolation techniques are well established (Kendal-Wright, 2007; Menon et al., 2013; Sato et al., 2016; Jin et al., 2018). However, although it is not impossible to isolate and culture primary chorion mesenchymal and trophoblast cells (CMC and CT) [99, 108], due to many *in vitro* challenges (isolation, culture conditions, passage-related issues, and transition properties), researchers have turned to use immortalized placenta-based trophoblast cells (i.e., BEWO and JEG-3) derived from carcinomas to replicate this layer [109]. As fetal membrane CTs reside in a functionally different region and perform distinct functions than placental trophoblast, the use of placental trophoblasts-derived cell lines to study chorionic membrane trophoblast functions are not ideal. The various cell types and cell sources that could be utilized to study the fetal membranes and feto-maternal interface are summarized in Table 1, together with their advantages and limitations.

#### *In vitro* Cell Culture Techniques

Two-dimensional (2D) single cell type culture experiments are most easy to run and low cost, and thus broadly utilized (Kendal-Wright, 2007; Menon et al., 2013; Meng et al., 2016; Sato et al., 2016; Feng et al., 2018b; Hadley et al., 2018; Jin et al., 2018). However, regardless of the cell origin (i.e., primary or immortalized) or cell layer (i.e., amnion or chorion), the major drawback is the limitations stemming from studying only a small part of the whole organ. Co-culturing two or more cell types allow researchers to study the organ system in a more holistic way, including studying cell–cell and cell–ECM interactions. Transwell co-culture is the current standard protocol to represent the amnion (i.e., AEC and AMC), amniochorion (i.e., AEC and CT or BEWO), and the feto-maternal interface (i.e., AEC and decidua) (Blanco et al., 2009; Talayev et al., 2010; Magatti et al., 2015; Wu et al., 2017; Lee et al., 2018; Richardson et al., 2019a). Despite providing a much more physiologically relevant model compared to 2D mono- or mixed culture, transwell culture has a variety of limitations, as summarized in Table 1.

#### *Ex vivo* Tissue Culture Techniques

Culturing fetal membrane tissues or biopsy-based explants obtained from discarded human fetal membrane from scheduled cesarean deliveries are commonly utilized for tissue-level culture and studies (Fortunato et al., 1994; Menon et al., 2011; Menon et al., 2014; Richardson et al., 2017a; Ayad et al., 2018). Additionally, live fetal membrane samples can be mounted into imaging chambers allowing for cellular and collagen visualization over time. Along with traditional explant treatments, these studies mimic biomechanical stressors, such a stretch, in order to delineate membrane weakening leading to rupture. These types of experiments enable a variety of molecular and biochemical assays using a more physiologically relevant model system and thus have significantly contributed to our current knowledge of fetal membrane physiology in the field (Fortunato et al., 1994; Miller and Loch-Caruso, 2010; Uchide et al., 2012; Boldenow et al., 2013; Menon et al., 2014; Kumar et al., 2016; Martin et al., 2017; Feng et al., 2018a; Hung et al., 2019). While explant culture maintains many advantages compared to other *in vitro* techniques, there are also many limitations, as summarized in Table 1.

## Co-Culture Organ-On-Chip Designs and Functions

Most organ systems are composed of two or more cell types that are arranged in a specific way to create various microarchitectures, where these multiple cell types closely interact and function together, giving rise to the unique structure and functions of each organ system (Bhatia and Ingber, 2014; An et al., 2015; Huh, 2015; van der Helm et al., 2016; Wilmer et al., 2016; Bein et al., 2018; Jodat et al., 2018; Sances et al., 2018). To recapitulate such complex multi-cellular structures, the majority of OOC systems (also called tissue chips or MPSs) require two or more different cell types to be co-cultured in specific arrangements. In addition, various interstitial flow (Artzy-Schnirman et al., 2019) and blood flow also directly access these multi-cellular architectures in a specific way (Ribas et al., 2016); thus, the multiple cell culture compartments of OOCs also need to be accessed by various fluids, creating distinct microenvironments for each cell types and cellular layers. In addition to cells, various ECMs secreted by cells are also significant components of most organ systems (Mondrinos et al., 2017; Pasman et al., 2018). Thus, OOCs have also to consider incorporating physiologically relevant ECMs. For all OOC systems, it is also essential to be able to monitor the cells and their microenvironment. Thus, being compatible with microscopy is critical. In addition, easy fluidic access to each cell layer, for both applying various biochemical stimuli and being able to analyze secreted metabolites, are also necessary. Here, we first provide a review of typical co-culture OOC architectures, and ECMs used, followed by examples of currently available OOC systems representing the feto-maternal interface.

### Co-culture OOC Architectures

**FIGURE 4:**
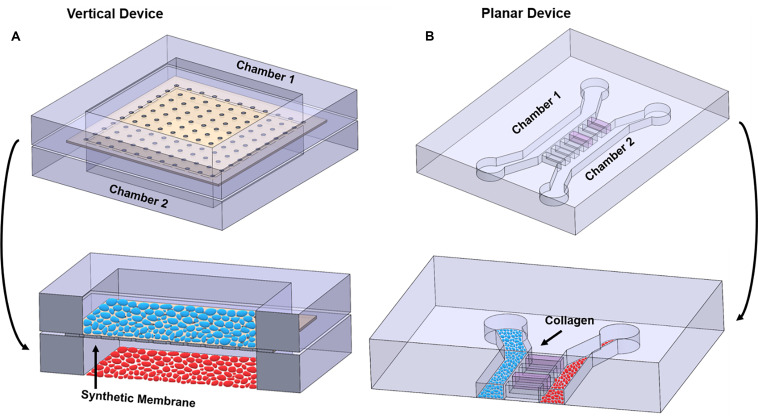
Illustration highlighting the differences between vertical and planar co-culture OOC designs. **(A)** 3D view of two cell culture chambers, stacked on top of each other, to form a “vertical co-culture” OOC device. These two chambers are separated by a semipermeable synthetic membrane (tan structure; black arrow), which contains small pores for cell migration and signal propagation, but too small for cells to freely move between the layers. Blue ellipses are grown on top of the membrane, while red ellipses are grown on the bottom glass substrate. Red and blue ellipses represent two distinct cell populations. **(B)** 3D view of two cell culture chambers aligned next two each other and separated by a set of microchannel arrays to form a “planar co-culture” OOC device. These two chambers are separated by microchannels that can be filled with collagen (pink; black arrow), providing an actual cell–collagen interface. Both blue and red ellipses are grown the bottom glass substrate but in separate chambers. Red and blue ellipses represent two distinct cell populations. The sizes of the microchannels are small enough to prevent cells from freely moving between the culture compartments but large enough for actively migrating cells and biochemicals to move between the compartments. Alternatively, these microchannel arrays can be replaced with a porous gel barrier.

Most co-culture OOCs fall under two design categories, “vertical” or “planar” co-culture designs, termed based on the orientation of the multiple microfluidic culture chambers that comprises the OOC (Figure 4). Each design has unique advantages and disadvantages, which are described below.

Multi-layered vertical co-culture OOCs are designed to contain vertically stacked cell culture chambers separated by a porous membrane (Figure 4A). Here, a semipermeable membrane separates the two vertically positioned cell culture chambers (Figure 4A; black arrow), allowing cells to be confined within each cell culture chamber while allowing biochemicals to flow through the membrane freely. This membrane often mimics the basement membrane layer and collagen, thus enabling cell–cell and cell–collagen interactions in the OOC environment (Pasman et al., 2018). The most commonly utilized membranes are the commercially available track-etched polyethylene terephthalate (PET) membrane, the same membrane utilized in transwell culture. These membranes come in varieties of different pore sizes, which can be selected to control the permeability between the cell culture compartments and can also mimic *in vivo* collagen density (i.e., pore size). These membranes can be coated with various ECMs collagens, partially recreating the cell–ECM interface. The two cell types can also be cultured on both sides of this membrane, minimizing the distances between the two cell types and allow better cell-cell interactions. Since these membranes are typically around 10 μm thick (Pasman et al., 2018) and made of plastic, and thus more rigid and thicker than what may be seen *in vivo* (i.e., 2 kPa and 13.4 ± 2.42 μm thick) (Halfter et al., 2013; Richardson et al., 2017b), custom membranes that are more thinner than commercially available ones have also been developed for OOC applications (Sip and Folch, 2014; Mondrinos et al., 2017; Pasman et al., 2018). The vertically positioned co-culture chambers can be fabricated with various materials (i.e., glass, polycarbonate, polyurethanes) and by multiple microfabrication processes (i.e., soft-lithography, laser engraving, CAD-based machining, and 3D printing), providing flexibility for various OOC applications. Overall, the vertical co-culture OOC design structurally mimics the *in vivo* structure, which is one of the major advantages of this design. Several OOC devices mimicking organs such as the lung, gut, and BBB utilize this design (Table 2) (Kim et al., 2012; Esch et al., 2016; Jeong et al., 2018).

**TABLE 2:** Examples of current co-culture OOC models that utilize vertical or planar co-culture designs.

**Lab group**	**Name of chip**	**Structure**	**Functionality**	**Cell types**	**Number of chambers**	**Journal name and year**
**Maschmeyer et al.**	Microfluidic four-organ chip	Planar	Human intestine, liver, skin and kidney co-culture to test drug metabolism	Human HepaRG cell line	6	Lab on a Chip, 2015
				Human primary hepatic stellate cell (HHSteC)		
				Human proximal tubule cell line (RPTEC/TERT-1)		
				Reconstructed human small intestinal barrier		
				Human juvenile prepuce		
**Carvalho et al.**	Colorectal tumor-on-a-chip	Planar	Precision nanomedicine delivery to a colorectal tumor	HCT-116 cancer cell	2	Science Advances, 2019
				Human colonic microvascular endothelial cell (HCoMEC)		
**Y. Guo et al.**	Biomimetic gut-on-a-chip	Planar	Drug metabolism in the intestine	Caco-2 cell	Four replicate chambers	Artificial Organs, 2018
**Gori et al.**	Non-alcoholic fatty liver disease-on-a-chip	Planar	Mimicking non-alcoholic fatty liver disease to understand how it can lead to hepatocellular carcinoma	Human hepatoma HepG2/C3A cell	2	PLOS One, 2016
**F. Yin et al.**	3D human placenta-on-a-chip	Planar	Establishment of 3D placental barrier and placental response to nanoparticle exposure *in vitro*	BeWo cell		Toxicology *in Vitro*, 2019
				Human choriocarcinoma cell	2	
				Human umbilical vein endothelial cell (HUVEC)		
**Jeong et al.**	3D blood–brain barrier model	Vertical	Blood–brain barrier function and permeability measurements	Primary astrocyte cell	2	IEEE Transactions on Medical Engineering, 2018
				Mouse brain endothelial cell (C57BL/6)		
**Esch et al.**	Modular body-on-a-chip	Vertical	Model drug metabolism in the GI tract epithelium and 3D primary liver tissue	Caco-2 Cell		Lab on a Chip, 2016
				Non-parenchymal cell (NPC)	2	
**Kim et al.**	Human gut-on-a-chip	Vertical	Mimic the flow and microbial flora environment of the gut	Caco-2 Cell	2	Lab on a Chip, 2012
				Human Caco-2 intestinal epithelial cell (Caco-2BBE)		
**Huh et al.**	Lung-on-a-chip	Vertical	Recreate the lung and test biological function	Human alveolar epithelial cell	2	Science, 2010
				Microvascular endothelial cell		

Despite these features and advantages, two significant limitations of this design are: (1) imaging of each cell culture compartment is relatively difficult due to the difficulty of imaging through the membrane that prevents imaging both cell types and (2) due to its multilayer design, microfabrication steps are more complicated and also reliable sealing of this sandwich structure is challenging. This also means that mimicking any organ structure that is composed of three or more cell layers, which is the case of the fetal membrane and feto-maternal interface, requires assembling multiple such sandwich structures, which pose even more challenges. This could significantly limit its applicability in many complex organ systems. In contrast, a planar co-culture design is ideal for cell visualization and fluid control because all the layers are on the same plane.

Planar co-culture OOCs are designed to contain parallel cell culture chambers separated by porous gel or microchannel arrays (Huh et al., 2011; Gumuscu et al., 2017; Richardson et al., 2019b), all in the same plane (Figure 4B). Here, the gel or microchannel array functions as a porous barrier that keeps cells within each cell culture chamber, while allowing various biochemicals to diffuse through. In the case of gel barrier-based systems, gel guiding microstructures (e.g., micropillar array or micro steps) are utilized so that the gel barrier fills only the space between the two culture chambers and prevents the gels that are being loaded to spill over and into the culture chambers (Vickerman et al., 2008; Chung et al., 2009; Funamoto et al., 2012; Osaki et al., 2020; Poussin et al., 2020). Here, the type of gel utilized, and its porosity determine how easily biochemicals can diffuse between the culture compartments. Another design strategy uses arrays of microfluidic channels that are small enough to prevent cells from moving from one compartment to the other (however, still allowing cell migration), but large enough for biochemicals to diffuse through. In this design, the length, size, and number of microfluidic channels control the degree of diffusion. These microfluidic channels can also be filled with various ECM components. In both cases, this gel barrier or microfluidic channel filled with ECM mimics a basement membrane and collagen layer that cells can actively degrade as seen *in vivo*, thus enabling cell–cell and cell–collagen interactions in the OOC environment (Richardson et al., 2019b; Osaki et al., 2020; Poussin et al., 2020). A major advantage of these planar designs is its compatibility and ease in microscopic imaging, as all structures are in the same focal plane and transparent. This makes it also ideal for identifying and monitoring cell–cell communication under different physiological environments, which is often challenging in vertical designs. The planar microfluidic designs are most commonly made out of polydimethylsiloxane (PDMS), but various other plastic that can minimize molecular adsorption can also be utilized (Berthier et al., 2012; Wang et al., 2012). These designs have been used extensively in OOC devices, such as those that mimic organs like the gut, liver, and multiorgan systems (Table 2) (Maschmeyer et al., 2015; Gori et al., 2016; Guo et al., 2018; Carvalho et al., 2019; Yin et al., 2019). Limitations of these planar designs also exist, such as difficulty in recreating tight junction barrier formation such as the BBB, and the larger distances between the co-culture compartments compared to the vertical co-culture OOC designs.

As summarized here, both co-culture OOC designs have been extensively utilized, with no one system being the perfect design, both having several advantages and disadvantages. This means that each OOC system design, even if mimicking the same organ system, must be decided based on what type of experiments researchers need to run and what kind of measurements are needed for such experiments. Understanding the “fit for purpose” concept when designing any OOC system becomes critical, as no one system can completely mimic the complex human organ system. In addition to these two designs, bioprinting of cells or scaffolds can also be utilized to create co-culture OOC devices (Miri et al., 2019; Mittal et al., 2019), but no such OOC devices of fetal membrane and feto-maternal interface exist as of yet, thus are not included in this review.

### *In vitro* Extracellular Matrices

In vertical co-culture OOC designs, synthetic membranes (e.g., track-etched PET membrane) are widely used to mimic the basement membrane (Sip and Folch, 2014; Mondrinos et al., 2017; Pasman et al., 2018) and regulate the communication between the two cell culture compartments. However, such synthetic membranes are quite different from the *in vivo* basement membrane, which is a key biological factor and plays many roles (Guller et al., 1995; Bryant-Greenwood, 1998; Strauss, 2013; Richardson et al., 2017a, b). Due to these reasons, synthetic membranes used in OOC systems are also often coated with various ECM materials to better mimic the *in vivo* environment (Pasman et al., 2018). However, this also makes it more challenging to control the porosity and diffusion characteristics, thus communication between the two cell layers. In planar co-culture OOC designs, gel barriers and microchannel array act as porous membranes. To better mimic the *in vivo* basement membrane, these microchannels can be filled with ECM materials (Bryant-Greenwood, 1998; Richardson et al., 2017b). Often, optimization of the type and concentration of ECMs loaded into this microchannel array is required to ensure the proper control over molecular diffusion and cell migration.

For OOCs to mimic the feto-maternal interface, co-culture OOC designs are a perfect candidate to recapitulate its microarchitecture and functionality under physiological and pathological conditions since the feto-maternal interface is composed of seven different cellular layers, some with ECMs and some without ECMs (van Herendael et al., 1978; Bryant-Greenwood, 1998; Avila et al., 2014; Richardson et al., 2017b; Menon et al., 2018). Such co-culture OOC models can mimic the feto-maternal interface and enable a better understanding of their physiology as well as how they are affected by drugs, toxicants, or other biochemical signaling factors.

### Fetal Membrane OOC (FM-OOC) Models

**FIGURE 5:**
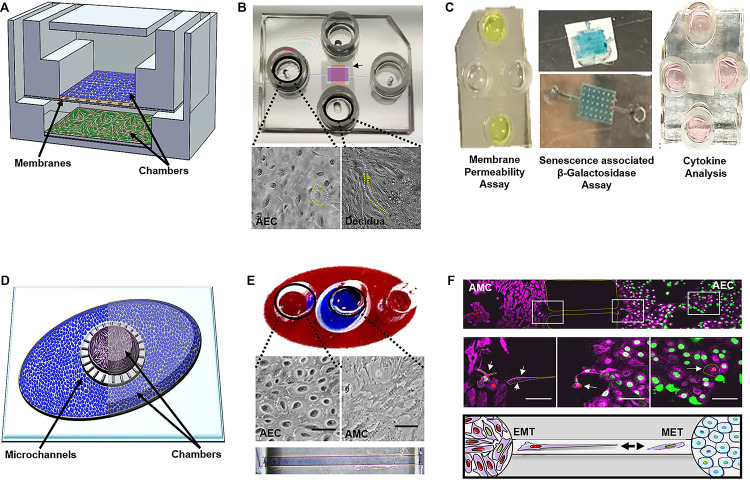
Currently developed OOCs mimicking components of the fetal membranes and feto-maternal interface. **(A)** Device layout—schematic of the two-chamber fetal membrane-organ-on-chip (FM-OOC) developed to study fetal and maternal cell interactions at the fetal membrane interface adapted from Richardson et al. (2019a). The FM-OO-C is comprised of two stacked PDMS cell culture chambers that are coated with Matrigel (pink). Primary AECs (blue) are placed in the top chamber and grown on top of a polycarbonate semipermeable synthetic membrane, while primary decidual cells (green) are placed in the lower chamber and grown on the glass substrate. **(B)** Images of the fabricated FM-OO-C chips and cells being cultured within each compartment. Bright-field microscopy images of primary human AECs in the top chamber (purple) and primary decidual cells in the bottom chamber (red) are shown. The yellow outline visualizes the cellular morphology. **(C)** Endpoint assays—Left: Fluorescein isothiocyanate stain (yellow) is seen in the two horizontal columns feeding into the top AEC chamber. Media were collected from the bottom vertical columns to measure membrane permeability. Center: Bottom chamber showing representative senescence-associated β-galactosidase (SA-β-Gal) stained decidual cells and the semipermeable membrane containing blue staining representing SA-β-Gal + AECs. Right: Image of the FM-OO-C containing media from both amnion and decidual cells, which can be used to measure cytokine kinetics. **(D)** Device layout—the amnion membrane organ-on-chip (AM-OOC) is designed to recreate the amnion component of the fetal membrane by co-culturing AECs (blue) in an outer circular PDMS chamber and AMCs (purple) in the inner circular chamber. This planar two-chamber model is separated by a type IV collagen-filled (pink) microchannel array (mimicking the basement membrane). **(E)** The outer chamber of the AM-OOC was filled with red dye, and the inner chamber was filled with blue dye for visualization. Bright-field microscopy images of AEC morphology and AMC morphology inside an AM-OOC device. Microchannels filled with Type IV collagen Matrigel (stained with Masson trichrome), connecting the two culture chambers, are also shown. **(F)** Endpoint assay—confocal images showing native AECs (green) and AMCs (red), which have transitioned, migrated, and integrated into the opposite population. Middle right panel highlights (yellow) GFP-AECs that have migrated through the type IV collagen-filled microchannel, re-localized vimentin, and transitioned into a mesenchymal morphology indicative of EMT. Middle left panels highlight (yellow) RFP-AMCs that have migrated through the type IV collagen-filled microchannel, down-regulated vimentin, and transitioned into an epithelial morphology indicative of MET. The bottom panel is a schematic representing AECs (green) and AMCs (red) undergoing cellular transitions. Gray arrows highlight the migration direction. Pink, vimentin; green, histone 2B AEC; red, histone 2B AMCs. This figure is a rendition of Richardson et al. (2019b). All figures reused with permission.

The first OOC to recreate components of the fetal membranes (i.e., the feto-maternal interface) was published in early 2019 (Richardson et al., 2019a). The FM-OOC was designed to better understand cellular interactions and paracrine cross-talk between maternal and fetal cells during pregnancy and parturition (Richardson et al., 2019a). The FM-OOC platform utilized the vertical co-culture OOC design and was composed of two orthogonal vertically stacked cell culture chambers containing equal surface areas. Primary AECs were seeded on top (fetal side), and primary decidual cells were placed on the bottom (maternal side). These chambers were separated by a semipermeable polycarbonate membrane (Figure 5A). The FM-OOC was utilized to detect membrane permeability, oxidative stress, and toxin-induced senescence, as well as cytokine production (Figure 5A). This device has many advantages over traditional transwell culture systems, including its ability to: (1) maintain physical and fluidic isolation between cell layers, (2) promote detectable biochemical changes, (3) better reproducibility, and (4) utilize fewer reagents and cells (Katt et al., 2016; Richardson et al., 2019a). However, it is still missing many vital components to recreate either the fetal membrane or the feto-maternal interface, such as (1) semipermeable membrane lacks ECM components to mimic that seen *in utero* (Richardson et al., 2019a), (2) organization of the device does not permit for imaging of both chambers, (3) direct imaging of migrating cells between chambers is not possible, where such cell migration is critical for understanding feto-maternal interface remodeling, and (4) lacks many cellular components of the feto-maternal interface, such as the AMCs, CMCs, and CTs (Richardson et al., 2017b, 2018b). Continuous advancement of FM-OOC models is expected, with the eventual goal to recreate the entire fetal membrane in OOC format. This device will be utilized to promote the study of cellular interactions during pregnancy and parturition, screening of drugs, and to advance research activities to reduce the risk of pregnancy-associated complications.

### Amnion Membrane OOC (AM-OOC) Models

To address the imaging limitations of the previously developed FM-OOC model described above, as well as focus more on the amnion membrane, which alone contains two cellular layers (Figure 2C), recently an amnion membrane OOC (AM-OOC) system was developed, the first of its kind (Richardson et al., 2019b). The AM-OOC system utilizes a planar parallel co-culture OOC model design, having two circular culture chambers with interconnected microchannel array in between that functions as a controlled permeable barrier between the compartments (Figure 5B) (Richardson et al., 2019b). By culturing primary human AECs in the outer circular chamber and AMCs in the inner circular chamber, separated by Type IV collagen-filled microchannels mimicking the basement membrane, they were able to recreate the amnion membrane on an OOC format (Richardson et al., 2019b) (Figure 5B). Here, primary AECs and AMCs obtained from the midzones of term not in labor fetal membranes were utilized. This model was successfully utilized to show the interactive and transitional properties of amnion cells (epithelial-to-mesenchymal transition and mesenchymal-to-epithelial transition; Richardson and Menon, 2018; Richardson et al., 2018a, 2020b) under normal and oxidative stress conditions, similar to how they behave and respond *in utero* (Richardson et al., 2019b) (Figure 5B). Although this planar device allowed for easy cell imaging of both compartments as well as direct monitoring and tracking of migratory cells between compartments, there were still aspects that did not fully recreate the amnion membrane. For example, the AMCs were cultured in 2D, while *in utero* they are embedded in 3D collagen. Also, the system did not contain all of the four cell types and layers of the fetal membranes.

## Perspective

### Proposed Ideas for a Full Feto-Maternal Interface OOC Model

**FIGURE 6:**
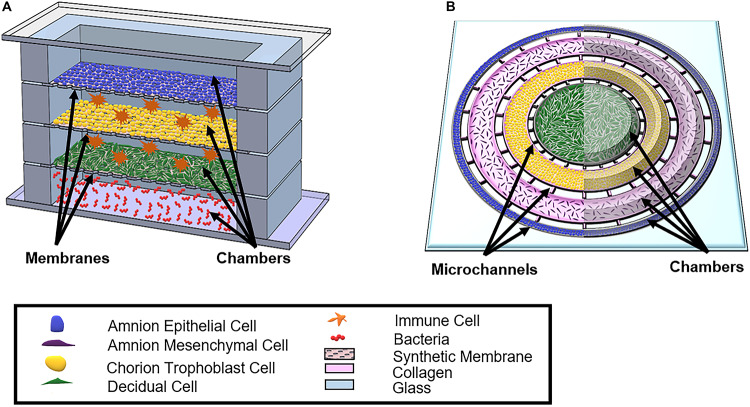
Schematic of proposed OOCs better mimicking the full fetal membrane and feto-maternal interface. **(A)** A rendition of the proposed fetal membrane on a chip (IFMOC) by Gnecco et al. (2017) designed to create an infectious preterm birth model to study fetal membranes. This device contains four chambers culturing AECs (blue) on top, CTs (yellow) along with immune cells (orange) in the second chamber, decidua (green) and immune cells in the third chamber, and bacteria (red) in the bottom chamber. Each chamber is separated by a polycarbonate semipermeable synthetic membrane. **(B)** The proposed feto-maternal interface organ-on-chip (FMI-OOC) here is designed to mimic the feto-maternal interface, including the fetal membranes and maternal decidua. The FMI-OOC contains four co-centric circular cell culture chambers separated by arrays of microchannels. The cells are seeded following the *in vivo* structure; AECs (blue), AMCs (purple), CMCs/CTs (yellow), and decidua cells (green), respectively. Primary fetal membrane collagen and Matrigel (pink) can enable culturing AMCs and CMC/CTs in a 3D format. To recreate cell–collagen interfaces, microchannels can be filled with type IV collagen (pink) to mimic the basement membrane of the amnion and chorion layers, while the choriodecidua interface is left open (gray). All figures reused with permission. A comparison of both proposed OOC models can be found in Table 3.

Although the field currently has two established co-culture OOC models (Richardson et al., 2019a,b) (Figure 5), significant advances need to be made to better study and understand the feto-maternal interface as a whole using an OOC model. Some advanced fetal membrane models have recently been suggested, although not developed yet, to better recapitulate the fetal and maternal side of the feto-maternal interface (Gnecco et al., 2017). The proposed device contains four vertical chambers, each chamber containing AECs, trophoblast cells, decidua, and bacteria (Gnecco et al., 2017) (Figure 6A). Here, with the help of media perfusion, immune cells (i.e., macrophages and leukocytes) are envisioned to be added to the chorio-decidua layers of this model to recreate an infectious preterm birth model (Gnecco et al., 2017) (Figure 6A). Although this design takes into consideration the cell density ratio seen *in vivo*, it does not contain amnion or chorion mesenchymal cells within the ECM of the fetal membranes, nor does it recreate any ECM components or cell–collagen interactions (Table 3). However, it does, for the first time, propose a four-chambered OOC model and discuss the importance of immune cell activation in preterm birth; both of these components deem this OOC novel and ahead of its time in 2017.

**TABLE 3:** Characteristics of the proposed OOCs compared to *in vitro* and *in vivo* conditions.

**Characteristics**	**Cell type**	**2D cell culture**	**3D cell culture**	**IFMOC**	**FMI-OOC**	**Mice models**	**Non-human primate**	**Human**
Morphology	AEC	Cuboidal/fibroblastoid	Fibroblastoid,	Cuboidal/fibroblastoid	Cuboidal/fibroblastoid	Cuboidal/fibroblastoid	Cuboidal/fibroblastoid	Cuboidal/fibroblastoid
	AMC	Fibroblastoid	Cuboidal/fibroblastoid,	Not present,	Fibroblastoid	Fibroblastoid	Fibroblastoid	Fibroblastoid
	CMC/CT	Fibroblastoid/cuboidal	Fibroblastoid/cuboidal	Only contain cuboidal CTs,	Fibroblastoid/cuboidal	Only contain cuboidal CTs,	Fibroblastoid/cuboidal	Fibroblastoid/cuboidal
	DECI	Fibroblastoid	Fibroblastoid	Fibroblastoid	Fibroblastoid	Not connected,	Fibroblastoid	Fibroblastoid
Collagen production	AEC	Low,	High	High	High	High	High	High
	AMC	High	High	High	High	High	High	High
	CMC/CT	Low,	High	High	High	High	High	High
	DECI	Low	Low	Low	Low	Low	Low	Low
Intermediate filament expression	AEC	Metastate	Mesenchymal	Metastate	Metastate	Metastate	Metastate	Metastate
	AMC	Mesenchymal	Metastate	Not present,	Mesenchymal	Mesenchymal	Mesenchymal	Mesenchymal
	CMC/CT	Mesenchymal/epithelial	Mesenchymal/epithelial	Only contain epithelial CTs,	Mesenchymal/epithelial	Only contain epithelial CTs,	Mesenchymal/epithelial	Mesenchymal/epithelial
	DECI	Mesenchymal	Mesenchymal	Mesenchymal	Mesenchymal	Not connected,	Mesenchymal	Mesenchymal

*Red font indicates differences.*

An alternative FM-OOC model proposed here would utilize a four-chamber planner co-culture OOC design, culturing primary AECs, AMCs, CMSs/CTs, and decidua cells (Figure 6B). Interconnecting each culture chamber can be an array of microchannels that are filled with ECMs, recreating the amnion and chorion basement membrane. Additionally, AMCs and CMCs/CTs can be suspended in Matrigel and/or decellularized amnion collagen, creating 3D cultures in these compartments (Figure 6B). Such a model would utilize OOC technology to recreate the microarchitecture of the feto-maternal interface down to every cell and collagen layer (Richardson et al., 2017b) (Figure 2C and Table 3). However, this device still lacks critical cellular components, including maternal and fetal immune cells, as well as the maternal layer of the decidua (parietalis). Integration of these cell layers along with biomechanical stressors (i.e., stretch) are needed in order to mimic the physiology of the feto-maternal interface.

Following the successful development of such a model, creating a pathologic condition of the feto-maternal interface, i.e., a disease OOC model, would be the next step. Such a disease model can mimic ascending and descending infection and inflammation, and be utilized to determine the propagation of infectious (e.g., lipopolysaccharides or bacteria) or inflammatory signals from maternal to fetal side, or vice versa, and test the efficacy of potential therapeutic compounds (i.e., anti-inflammatory molecules or synthetic drugs) in suppressing inflammation in each layer. Importantly, employing such a disease OOC model can contribute to the development of novel therapeutics against preterm birth, a very much needed area of developing considering that around 9.8% people in the United States alone are affected (Goldenberg et al., 2008; Liew et al., 2008; Beck et al., 2010; Blencowe et al., 2013; Lawn et al., 2013) while having the potential to significantly reduce the time and cost associated with pre-clinical and clinical trials. However, like any other model system, the developed OOCs also have several limitations, including: (1) each OOC is designed to answer certain biological questions, limiting their universal use, (2) requirements for specialized equipment to fabricate and conduct experiments, although this is not becoming easier, (3) have the tendency to be lower throughput, and (4) multi-organ chips are not available to model pregnancy.

### Next Steps for Pregnancy-Related *in vitro* Methodologies

Although OOCs relating to the field of obstetrics are emerging over the past 5 years (Blundell et al., 2016, 2018; Lee et al., 2016; Gnecco et al., 2017; Nagashima et al., 2018; Pemathilaka et al., 2019a, b; Richardson et al., 2019a, b; Yin et al., 2019), significant future research is needed in order to truly create an *in vitro* pregnancy model to better understand feto-maternal communication, the induction of term and preterm labor, and drug or toxicant permeability at these vital interfaces. Advances from traditional 2D culture systems to novel 3D culture platforms are contributing to overcoming these knowledge gaps. 3D cell culture, typically referred to as an organoid culture [i.e., cell spheroids (Okere et al., 2015), cell sheets (Richardson et al., 2020a), or tissue printing (Kang et al., 2016)], utilizes cell aggregates either with single cell type or multiple cell types, often together with various ECMs, to recreate components of the fetal membrane and feto-maternal interface (Liu and Qi, 2010; Davydova et al., 2011; Shieh et al., 2017). While 3D growth of cells has been documented in many fields to induce expression of more *in vivo* characteristics and functionality, only a handful of studies have been conducted with fetal membrane-derived cells. Importantly, no attempts have been made so far to recreate the fetal membrane or feto-maternal interfaces using such 3D bioprinting techniques that have been utilized to print volumetric shapes of cell and collagen layers to recreate ear, noses, and eye components (Kuru et al., 2016; Isaacson et al., 2018; Jodat et al., 2020). The advantages and limitations of each of these 3D culture techniques are also summarized in Table 1.

### Impact to Clinical Research

Although many clinical studies have been conducted evaluating different aspects of fetal membranes, it is still challenging under certain settings to acquire approval and or recruit enough patients within individual clinical conditions (i.e., preeclampsia, pPROM, chorioamnionitis, gestational diabetes) in order to provide tissue for basic research and/or to conduct clinical trials [162, 163]. A “pregnancy-on-chip” platform that can represent various pathologic conditions of pregnancy could provide a useful model to conduct clinical trials that generally could not occur. This model is also ideal for testing FDA-approved drugs that currently do not contain enough pre-clinical data related to transport across the feto-maternal interfaces. This cost-effective approach could lead to the approval of dozens of drugs to be repurposed toward treating pregnancy-related complications. Additionally, OOC-based studies can be adapted to clinical research to conduct experiments that can lead to better understand the mechanism of drug functions (e.g., efficacy, cytotoxicity, passage through distinct layers of the feto-maternal interfaces), and be utilized for pre-clinical trials of therapeutic development against preterm birth, or even replace part of a clinical trial.

Besides, the recent European Union (EU) ban on animal testing for cosmetic products (No Author, 2013), as well as US EPA’s current directive that prioritizes efforts to reduce animal testing, are expected to further spur this area. The development of novel OOC models suggests two new promising concepts: (1) “personalized medicine-on-chip” by using patient-derived cells, including primary cells and inducing patient-derived fat cells into iPSCs, which can consider the effect of patient-to-patient variability (Jodat et al., 2018; van den Berg et al., 2019) and (2) applying the “human-on-chip” concepts to clinical trials (Luni et al., 2014; Abaci and Shuler, 2015; Maschmeyer et al., 2015).

### Impact on Basic Research

The many challenges in this area are not just faced clinically but also while conducting basic biological research. Current, *in vitro* (i.e., 2D, 3D, or transwell culture) and *ex vivo* (i.e., explant culture) assays provide complexity in understanding multi-organ communication between individual cellular or collagen layers within the tissue and or organ-to-organ systems (Tables 1, 3). Understanding this communication is vital to answering physiological questions related to gestation, term, and preterm parturition, as well as the pharmaceutical questions related to pre-clinical trials. Furthermore, understanding individual cellular contribution to labor onset and adverse pregnancy outcomes could potentially identify novel biomarkers of term and preterm delivery. Biomarkers identified in this manner could lead to the development of standard clinic testing for patients having a risk for preterm labor.

## Conclusion

Organ-on-chips represent a variety of physiological and pathophysiological states of diverse organ structures that are contributing to a better understanding of complex organ systems. These platforms also have the potential to become critical steps in the drug discovery pipeline, as well as part of the future of bench to bedside research. Although the importance of the feto-maternal interface in pregnancy has been documented for decades, only recently has the technology allowed for novel *in vitro* techniques to recreate the anatomy and function of the placenta and fetal membranes accurately. Several placenta-on-chip (Blundell et al., 2016, 2018; Lee et al., 2016; Pemathilaka et al., 2019a, b; Yin et al., 2019) and fetal membrane-on-chip (Richardson et al., 2019a, b) platforms have emerged by mimicking the microarchitecture and functions of both feto-maternal interfaces, and are improving our understanding of this vital organ system. As more advanced OOC models of the feto-maternal interface emerge, we expect such models to radically change how research and development are conducted in the field of obstetrics.

## Author Contributions

LR and SK drafted the manuscript and created the figures, while AH and RM reviewed and edited the manuscript. All authors contributed to the article and approved the submitted version.

## Conflict of Interest

The authors declare that the research was conducted in the absence of any commercial or financial relationships that could be construed as a potential conflict of interest.
